# CD-1 Outbred Mice Produce Less Variable Ultrasonic Vocalizations Than FVB Inbred Mice, While Displaying a Similar Developmental Trajectory

**DOI:** 10.3389/fpsyt.2021.687060

**Published:** 2021-08-13

**Authors:** Matthew S. Binder, Hannah D. Shi, Angelique Bordey

**Affiliations:** Department of Neurosurgery and Cellular and Molecular Physiology, Yale School of Medicine, New Haven, CT, United States

**Keywords:** USV, behavior reproducibility, neurodevelopmental disorders, methods, communication, neonatal vocalization

## Abstract

The production of ultrasonic vocalizations (USVs) in neonatal mice is a critical means of communication that is used to elicit maternal care. Alterations in neonatal USV production is also an indicator of neurological deficits. However, USVs have been predominately assessed in inbred animals and are significantly understudied in outbred mice, even though outbred animals better represent the genetic diversity of humans and are used in several neurological disorder models. To determine the reproducibility of USVs across models, we compared male and female CD-1 (outbred) and FVB (inbred) mice on postnatal days (PD) 4, 8, 12, 16, and 20. We found that CD-1 and FVB mice displayed a similar developmental trajectory of USVs. However, CD1 mice emitted more USVs on PD 12 than FVB mice. In addition, FVB mice emitted a longer duration of calls on PD 4 and 8 and a higher overall maximum and minimum frequency of USVs than CD-1 mice. No differences in mean amplitude were found between groups. We also detected numerous significant differences between outbred and inbred mice when comparing each group's call composition. We next assessed the relative variability of mouse vocalizations between groups, finding that outbred mice were less variable than inbred mice. For the spectral and temporal characteristics of the USVs, variability was similar between groups. Altogether, we found that CD-1 outbred mice display a similar, if not lower, degree of variability than FVB inbred mice when assessing neonatal USVs.

## Introduction

Vocal communication is a constituent of various species including mice, songbirds, dogs, dolphins, and humans (1–4). Producing vocalizations serves many purposes as they are used in neonates to elicit maternal care and are also used in adults to mark the presence of food, predators, or territory, and to assert social status and reproductive interest (5–9). However, vocalizations are also a reliable indicator of an animal's overall health, with disruptions in vocalizations characterizing numerous neurological diseases. Specifically, altered vocalizations have been found in neurodegenerative disorders such as Alzheimer's disease and frontotemporal dementia, as well as in neurodevelopmental conditions such as autism spectrum disorder, tuberous sclerosis complex, epilepsy, and Tourette's syndrome (10–15). Importantly, these altered vocalizations in disease states are highly conserved across species, as they are observed in both clinical settings and in preclinical models, further reinforcing their pertinence to, and utility in, neurological conditions (3, 11, 13, 16–20). Therefore, vocal communication not only encompasses an expansive and vital behavior, but one that is highly relevant to human health.

The unique implications of vocal communication have been studied *via* the use of murine models. Vocal communication in mice refers to the production of ultrasonic vocalizations (USVs), which are whistle-like calls emitted between 30 and 90 kHz (21). USVs can be emitted across the lifespan, with pups emitting vocalizations in order to elicit maternal retrieval and adults vocalizing during mating behaviors however, USVs have been most comprehensively and commonly assessed in neonates, due to the consistency of neonatal USVs and the relative ease of their paradigm (22, 23). To date, the majority of both neonatal and adult USV research has used inbred mice due to their genetic stability and limited mouse to mouse variation (24). However, preferentially assessing inbred mouse vocalizations is potentially problematic. Outbred mice, by definition, are genetically complex, which in turn better resembles the complexity of the human genome and therefore may yield results that have a greater generalizability (25, 26). Furthermore, Tuttle et al. (27) assessed trait stability in inbred and outbred mice using 26 measures and found compelling evidence indicating that in most cases (20 out of 26) outbred mice are as variable, if not less variable, than inbred mice, counteracting a perceived strength of the inbred model. Moreover, there is evidence to suggest that more genetically diverse animals are less sensitive to changes in environmental and experimental conditions than their inbred counterparts, indicating that results may be more consistent, and thus more reproducible, across studies utilizing outbred mice (27, 28). However, despite the apparent advantages of outbred mice, few studies have investigated outbred mouse USVs and no study has comprehensively characterized the developmental trajectory of vocalizations in outbred mice and directly compared the USV profiles of neonatal inbred and outbred mice (29, 30).

Due to the importance of communicative behaviors and their pertinence to disease states and implications for human health, it is essential to know if inbred vocalizations, which constitute the majority of the literature, resemble the vocalizations of the more generalizable outbred mice. Furthermore, characterizing the developmental trajectory of outbred vocalizations would not only provide additional context for existing USV studies, but would also contribute to a foundation that other outbred studies could build upon. Therefore, to address these needs, our study assessed the vocalizations of CD-1 outbred mice throughout the neonatal period and compared them to FVB inbred mice from the same background strain.

## Materials and Methods

### Animals and Housing

CD-1 outbred mice and FVB inbred mice (both originally derived from Swiss mice) were purchased from Charles River. The strain of a mouse has been shown to significantly affect the quantity of USVs produced, with some strains innately producing more vocalizations than others (3, 31). In order to minimalize strain dependent variance (to the extent that it is possible when comparing outbred and inbred animals), we used mice that were derived from the same background (Swiss). A total of 60 mice were tested in this study coming from 16 different litters: 15 male CD-1, 15 female CD-1, 15 male FVB, and 15 female FVB mice. This sample size was determined by an a priori power analysis. Mice were toe clipped on PD 4 after USV assessment which allowed specific mice to be identified throughout the course of the study. Mice were weighed following behavioral assessment at each timepoint. All animals were tested during the light cycle between 9 a.m. and 2 p.m. The mice were kept in a climate-controlled colony room on a 12-h light/dark diurnal cycle and given *ad libitum* access to food and water. All test procedures were carried out in compliance with the National Institutes of Health Guidelines for the Care and Use of Laboratory Animals and were approved by Yale University's Institutional Animal Care and Use Committee.

### Neonatal Ultrasonic Vocalizations

In order to garner a thorough understanding of the USV profile of outbred mice relative to inbred mice, we assessed vocalizations at 5 timepoints: PD 4, 8, 12, 16, and 20. These timepoints were chosen to be in accordance with other (inbred) vocalization development characterization studies (3, 31). Furthermore, while studies have shown that the strain of the mouse may affect the quantity of USVs produced, it has been well-established that virtually all neonatal inbred vocalizations follow a similar trajectory, with USVs increasing after birth, typically reaching a peak around days 7–9 and decreasing significantly at PD 14 (21, 32). Therefore, our selected timepoints encompass the typical trajectory of neonatal vocalizations, allowing for a comprehensive comparison.

USVs were elicited via the maternal separation paradigm which has been previously described (3, 33). Briefly, pups were habituated to a 22 °C testing room for 30 min prior to the testing period. The pups were then separated from their dam and placed into a clean housing cage preheated to an ambient nesting temperature (~35 °C). Next the pups were individually removed from the housing cage and placed into a clean test cage contained within a 30 × 30 × 20 cm sound attenuating acrylic chamber. Ultrasonic vocalizations were recorded for a 2-minute duration using a broad-spectrum condenser microphone with a range spanning 1–125 kHz (CM16/CMPA, Avisoft Bioacoustics, Glienicke, Germany part #40011) and a recording interface (UltraSoundGate 116Hb, Avisoft Bioacoustics part # 41161/41162), in accordance with prior studies (18, 33). The microphone was suspended above the center of the cage, making the distance between each pup and the microphone approximately 7 inches. The pups were not restrained and were thus allowed to freely move for the duration of the trial. Upon completion of the trial, the test mouse was removed from the test chamber. Once testing concluded for all mice, they were returned to their home cage.

### DeepSqueak Analysis

DeepSqueak analysis was conducted as previously described (34). Specifically, DeepSqueak was downloaded from Github and accessed via Matlab 2018a software. The.wav USV files were imported into DeepSqueak and the total analysis length was set to 0, the analysis chunk length to 6, the frame overlap to 0.0001 s, the frequency low cut off to 30 kHz, the frequency high cut off to 120 kHz, and the score threshold to 0. The detection parameters were set to “high recall” to ensure that all of the USVs present were detected. The files were then manually processed, with the tonality threshold being adjusted to optimize the signal-to-noise ratio for each file and the automatic detection boxes being redrawn as needed in order to accurately and consistently detect the spectral and temporal characteristics of the vocalizations. The call type composition per each strain was assessed by manually going through each file and labeling the detected calls. Specifically, the Scattoni call type taxonomy (2011) was used to identify the vocalizations and sort them into 1 of 10 discrete categories based off of internal pitch changes, call length, and call shape (3, 35).

### Statistical Analysis

All data was analyzed using IBM SPSS Statistics 21.0 (IBM, USA) or GraphPad Prism 7 software (La Jolla, CA). The differences in the quantity of vocalizations between outbred and inbred mice across timepoints were analyzed with a repeated-measures ANOVA, with group and sex as between-subjects factors and the USVs emitted on PD 4, 8, 12, 16, and 20 as the within subjects variables. A similar analysis was run to analyze the differences in the average duration, minimum frequency, maximum frequency, and the mean amplitude (loudness) of the calls. The above parameters were chosen since each has been shown to be of particular relevance to communicative behaviors as a whole, as well as to atypical communicative behaviors in neurological conditions (3, 11, 13, 36–38). All interactions were clarified using the Tukey HSD post hoc analysis. The call type composition of each group was analyzed with a Pearson Chi-Square, along with individual z-tests, to compare significant call type proportions between groups. Call type composition was assessed on PD 8 to be in alignment with other studies and to maximize our study's points of comparison (3, 39). The relative dispersion of the USVs emitted per each timepoint between inbred and outbred mice were assessed via calculating the mean and standard deviation of the groups, as well as the coefficient of variability (CV), as this has previously been used to compare the behavioral variance between outbred and inbred animals (27). Furthermore, the weights of the mice in both groups were compared via a repeated measures ANOVA. A value of *p* < 0.05, was considered significant for each statistical test, with figures depicting the mean ± standard error of the mean (SEM).

## Results

### Ultrasonic Vocalization Developmental Trajectory

**Figure 1:**
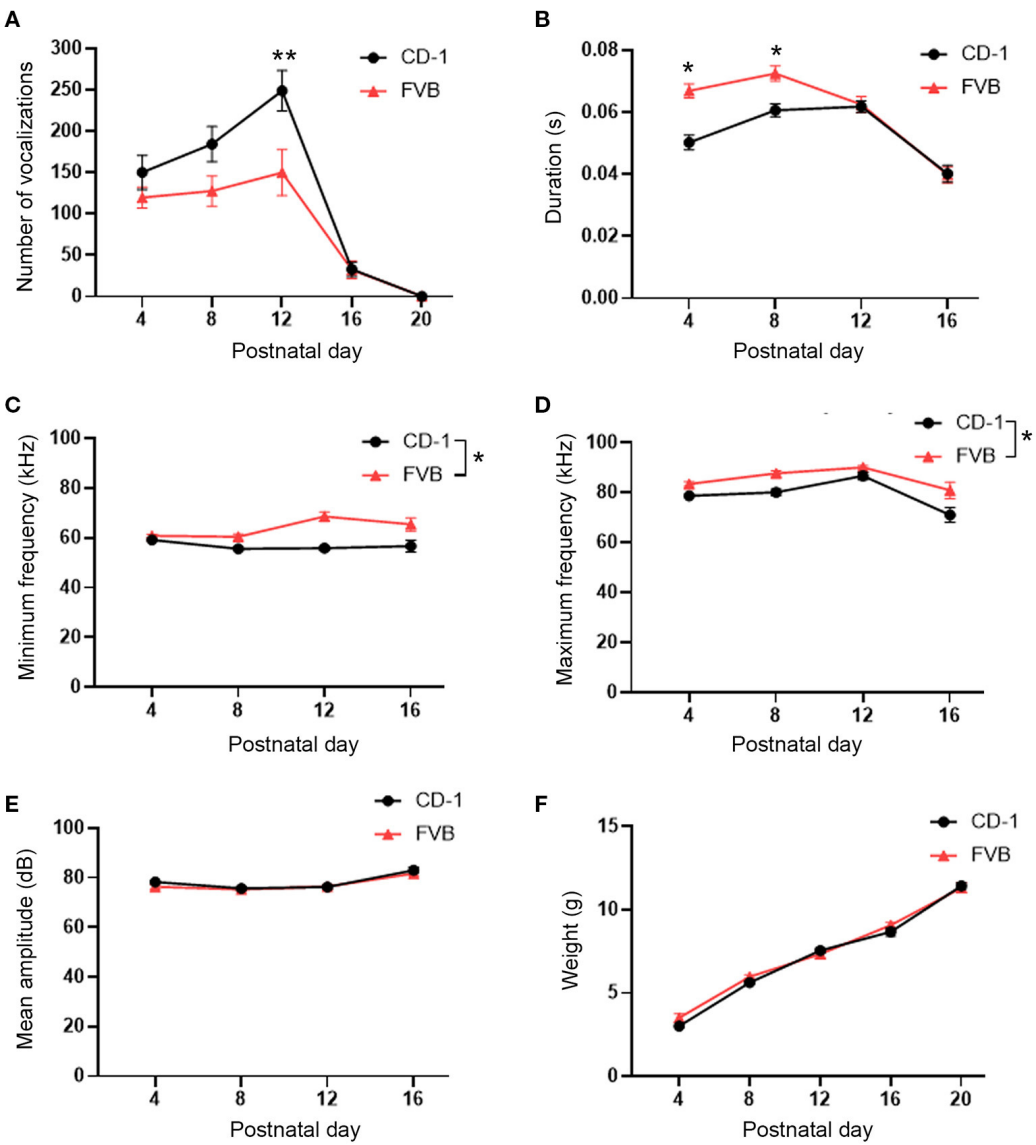
USV developmental trajectory and comparison in CD-1 outbred and FVB inbred mice. **(A)** CD-1 and FVB mice display a similar USV development, however, CD-1 mice emitted significantly more USVs on PD 12 than FVB mice emitted on PD 12, with no other differences present between groups. **(B)** FVB mice emitted USVs of a shorter duration than CD-1 mice on PD 4 and 8, with no other differences present. **(C)** FVB mice emitted USVs of a higher overall minimum frequency than CD-1 mice. **(D)** FVB mice emitted USVs of an overall higher maximum frequency than CD-1 mice. **(E)** There were no differences in mean amplitude between CD-1 and FVB mice. **(F)** Animal's weights increased linearly overtime, with no differences between groups present. A total of 60 mice were assessed, 15 males and females per each group. The data points represent the mean and the error bars represent the standard error of the mean. *p < 0.05, **p < 0.01.

Vocalization production was assessed over time between two groups, CD-1 outbred and FVB inbred mice, using a repeated measures ANOVA. Thirty mice (15 male and 15 female) were examined per group. When comparing male and female USV production, there was no main effect present for sex, nor any group by sex interaction, and no day by sex by group interaction (data not shown, *F* and *p*-values in Supplementary Table 1). As a result, data from male and female mice were pooled. We then found that there was a main effect of group, a day by group interaction, and a main effect for the within subjects variable of day (*F*_1, 56_ = 6.24, *p* = 0.02, *F*_4, 224_ = 3.19, *p* = 0.01, and *F*_4, 224_ = 46.20, *p* < 0.001, respectively). *Post hoc* tests found that, for CD-1 outbred mice, there was no difference in the quantity of USVs produced on PD 4 vs. PD 8. However, on PD 12, CD-1 mice emitted significantly more USVs than at any other timepoint. CD-1 mice also emitted significantly fewer USVs on PD 16 than the preceding timepoints, with no USVs being emitted on PD 20. For FVB mice, there was no significant difference in USVs emitted for PD's 4, 8, and 12. However, on PD's 16 and 20 FVB mice emitted significantly fewer USVs, with no USVs again being emitted on PD 20. When comparing across groups, CD-1 mice emitted significantly more USVs than FVB mice on PD 12 (*p* < 0.05), however, there were no significant differences between groups for PD 4, 8, 16, and 20 (Figure 1A).

### Trajectory of USVs' Spectral and Temporal Characteristics

We next assessed USV characteristics, including duration, minimum frequency, maximum frequency, and amplitude. For all USV characteristics, there was no effect of sex, no day by sex, or group by sex interactions, and no day by sex by group interaction (Supplementary Table 1). Female and male data were then pooled. For duration, we observed a main effect of group, an interaction between group and day, and a main effect for the within subjects variable of day (*F*_1, 39_ = 10.02, *p* < 0.01, *F*_3, 117_ = 4.39, *p* < 0.01, and *F*_3, 117_ = 28.01, *p* < 0.001, respectively). *Post hoc* analysis revealed that CD-1 mice emitted shorter mean USVs on PD 4 (*p* < 0.05), with USVs increasing and plateauing on PD 8 and 12 (*p* > 0.05) before significantly decreasing on PD 16 (*p* < 0.05). Meanwhile, FVB mice emitted a shorter duration of USVs on PD 4 (*p* > 0.05), with USV duration increasing on PD 8 (*p* > 0.05) then decreasing on PD 12 (*p* > 0.05), with USVs being at their shortest on PD 16 (*p* < 0.05). When comparing CD-1 to FVB mice, we found that CD-1 mice emitted USVs of a significantly shorter duration on PD's 4 and 8, with no differences between groups on PD's 12 and 16 (*p* < 0.05) (Figure 1B).

When assessing the minimum frequency, there was a main effect of group (*F*_1, 39_ = 35.99, *p* < 0.001), with CD-1 mice emitting a lower minimum frequency than FVB mice (Figure 1C). There was no day by group interaction and no main effect for the within subjects variable of day (*F*_3, 117_ = 2.03, *p* = 0.11 and *F*_3, 117_ = 2.22, *p* = 0.09, respectively).

When assessing the maximum frequency of USVs, there was a main effect of group (*F*_1, 39_ = 14.10, *p* = 0.001), with CD-1 mice emitting an overall lower maximum frequency than FVB mice (Figure 1D). There was no day by group interaction (*F*_3, 117_ = 0.86, *p* = 0.46). There was a main effect for the within subjects variable of day (*F*_3, 117_ = 12.65, *p* < 0.001). Corresponding assessments revealed that the highest maximum frequencies were on PD 12 (*p* < 0.05), with the lowest maximum frequencies being emitted on PD 4 (*p* < 0.05) and PD 16 (*p* < 0.05).

For USV amplitude, there were no main effects of group and no day by group interaction (*F*_1, 39_ = 0.18, *p* = 0.67 and *F*_3, 117_ =.61, *p* = 0.61). There was a main effect for the repeated measures variable of day (*F*_3, 117_ = 15.83, *p* < 0.001). Further tests revealed that vocalizations had the highest amplitude on PD 16 (*p* < 0.05) (Figure 1E). Therefore, while there was only a difference at one timepoint between outbred and inbred mice for USV production, there are numerous differences across many timepoints between outbred and inbred mice when assessing the spectral and temporal characteristics of USVs.

### Call Type Composition Analysis

**Figure 2:**
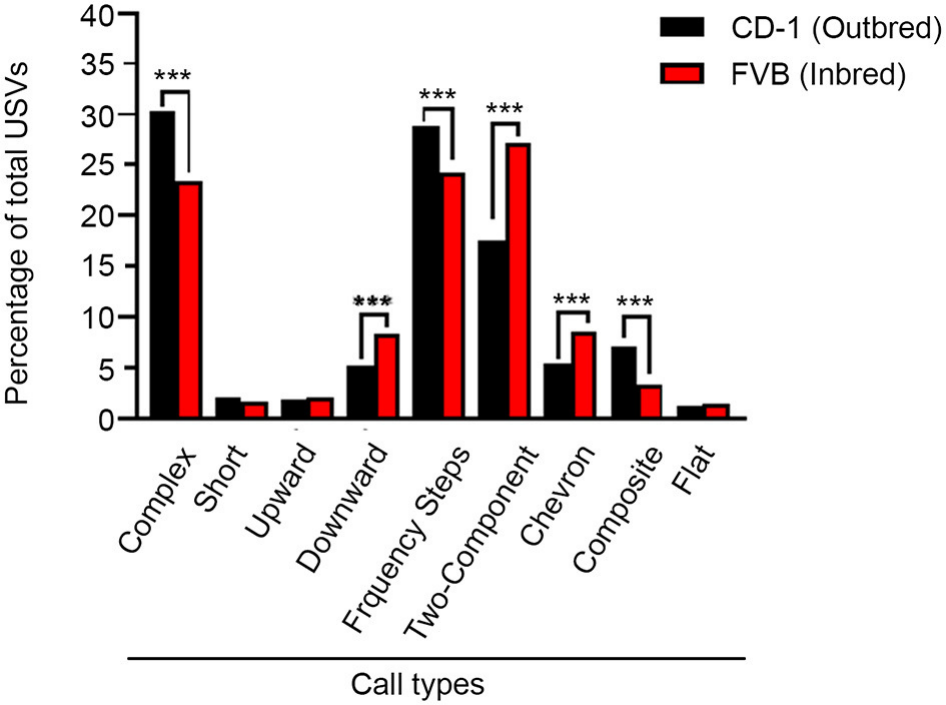
Call type composition for CD-1 and FVB mice. PD 8 CD-1 mice emit more complex, frequency steps, and composite call types than FVB mice but less downward, two-component, and chevron call types. No differences between groups were found for short, upward, and flat call types. ***p < 0.001.

In addition to assessing the quantitative characteristics of vocalizations, we also assessed qualitative features of the calls. When examining the call types, a Pearson Chi-Square analysis revealed significant group differences between the composition of calls for CD-1 and FVB mice (X${}_{8,\text{N}=7707}^{2}$ = 203.69, *p* < 0.001). Proportional differences detected with z-tests found that CD-1 animals emitted a significantly greater quantity of complex, frequency steps, and composite call types, than FVB mice. CD-1 mice also emitted significantly fewer downward, two-component, and chevron call types, with no differences between groups for short, upward, and flat call types (Figure 2).

### Weight Assessment Across Timepoints

The weight of a pup may be a factor that differentially affects USV production (40). To rule out this potential confound, the weights of CD-1 and FVB mice were directly compared across each timepoint. A repeated measures ANOVA was run and there was no main effect of group and no day by group interactions (*F*_1, 56_ = 0.64, *p* = 0.43 and *F*_4, 224_ = 3.48, *p* = 0.07, respectively) There was also no effect of sex for any parameters (Supplementary Table 1). However, there was a main effect of day (*F*_4, 224_ = 518.96, *p* < 0.001). Tukey's test was preformed to further assess the data and found that the weight of the mice was lowest on PD 4. It also found that the weights of the mice on PD's 8, 12, 16, and 20 were all significantly higher than the preceding timepoints (*p* < 0.05) (Figure 1F). Therefore, the lack of difference in weight between CD-1 and FVB mice indicates that the size of the mice did not differentially affect vocal production.

### Variability Assessment for the Quantity of Vocalizations

We next assessed the inherent variability of inbred and outbred USVs. The mean, standard deviation, and coefficient of variability were calculated per each timepoint and group and are listed in Table 1 (the lower CV value between groups is bolded). We found that on PD 4, FVB mice were less variable in their vocal production than CD-1 mice. However, on PD 8, 12, and 16, CD- 1 mice exhibited less variability than FVB mice. We also combined the data and assessed the total variability between groups. We found that CD-1 mice were significantly less variable than FVB mice (>10% difference of CV between groups). Therefore, CD-1 mice emitted a less variable quantity of vocalizations than FVB mice on both a per day basis and overall.

**Table 1:** Variability in USV production per day and in total between groups.

	**Ultrasonic vocalization quantity**					
	**CD-1**			**FVB**		
	**CV**	**x̄**	**SD**	**CV**	**x̄**	**SD**
PD 4	75.4	150.1	113.1	57.3^*^	119.5	68.5
PD 8	77.9^*^	127.6	99.4	80.4	126.0	101.2
PD 12	53.9^*^	248.8	134.1	101.8	149.8	152.5
PD 16	146.7^*^	32.8	48.2	182.1	32.1	58.5
Total	91.6^*^	139.8	128.1	103.2	110.2	110.3

* *The values with lower variability between groups are bolded. CV, coefficient of variability; x̄, mean; SD, standard deviation*.

### Variability Assessment of the Spectral and Temporal Characteristics of Vocalizations

The mean, standard deviation, and coefficient of variability for the spectral and temporal characteristics of USVs are depicted in Table 2. For duration, FVB mice were slightly less variable than CD-1 mice on PD 4 and PD 16. However, CD-1 mice were slightly less variable than FVB mice on PD 12. On PD 8 there was a similar degree of variability between groups. When the variability of the total duration was compared, we found that FVB mice were slightly less variable than CD-1 mice. Therefore, there was minimal variability per day (<6% difference of CV) and overall (<1% difference of CV) between groups when assessing duration (Table 2).

**Table 2:** Variability in USV characteristics per day and in total between groups.

	**Duration**						**Minimum frequency**					
	**CD-1**			**FVB**			**CD-1**			**FVB**		
	**CV**	**x̄**	**SD**	**CV**	**x̄**	**SD**	**CV**	**x̄**	**SD**	**CV**	**x̄**	**SD**
PD 4	25.6	0.050	0.013	18.9^*^	0.067	0.013	13.3	59.2	7.9	5.9^*^	83.5	5.0
PD 8	18.8	0.061	0.011	18.4^*^	0.073	0.013	7.9^*^	55.7	4.4	10.9	60.4	6.6
PD 12	16.5^*^	0.063	0.010	20.2	0.061	0.012	12.8	56.9	7.3	11.7^*^	68.2	8.0
PD 16	34.1	0.040	0.014	28.4^*^	0.040	0.011	20.8	56.7	11.8	16.8^*^	80.9	13.6
Total	27.4	0.054	0.015	27.1^*^	0.062	0.017	14.1	57.1	8.1	12.4^*^	63.3	7.8
	**Maximum frequency**						**Mean amplitude**					
	**CD-1**			**FVB**			**CD-1**			**FVB**		
	**CV**	x̄	**SD**	**CV**	x̄	**SD**	**CV**	x̄	**SD**	**CV**	x̄	**SD**
PD 4	10.8	78.7	8.5	5.6^*^	60.9	3.4	5.0^*^	78.3	3.9	6.1	76.4	4.7
PD 8	9.5	80.1	7.6	6.7^*^	87.7	5.9	5.2^*^	75.7	3.9	5.2^*^	75.7	3.9
PD 12	9.9	86.6	8.6	3.7^*^	90.4	3.3	6.4	75.9	4.9	4.0^*^	77.0	3.1
PD 16	20.8	71.1	14.8	16.6^*^	65.3	10.8	7.5	83.1	6.2	4.2^*^	82.2	3.5
Total	14.2	79.5	11.3	9.3^*^	85.9	8.0	7.0	78.0	5.5	6.0^*^	77.2	4.6

* *The values with lower variability between groups are bolded. CV, coefficient of variability; x̄, mean; SD, standard deviation*.

When assessing the minimum frequency of the USVs elicited, we found that FVB mice were slightly less variable than CD-1 mice on PD 4, 12, and 16, however, CD-1 mice exhibited less variability than FVB mice on PD 8. When assessing the total variability between groups, FVB mice were slightly less variable than CD-1 mice. Thus, there was minimal variability per day (1–8% difference of CV) and overall (2% difference of CV) between groups (Table 2).

For maximum frequency, FVB mice were slightly less variable than CD-1 mice on each day. The total variability for maximum frequency between groups was also similar, with FVB mice being slightly less variable than CD-1 mice. Thus, there was limited variability for maximum frequency between groups per day (<6% difference of CV) and overall (<5% difference of CV) (Table 2).

Lastly, for mean amplitude, CD-1 mice were slightly less variable than FVB mice on PD 4. However, FVB mice were less variable than CD-1 mice on PD 12 and 16, and both groups exhibited the same variability at PD 8. When assessing the overall mean amplitude, we found similar variability between groups. Therefore, for mean amplitude, there was minimal variation between CD-1 and FVB mice per day (<3% difference of CV) and overall (1% difference of CV). Thus, across all spectral and temporal parameters, CD-1 and FVB mice displayed similar variability.

## Discussion

The present study characterized the developmental trajectory of CD-1 outbred mouse vocalizations and compared them to FVB inbred mice in order to elucidate the role genetic complexity may have on neonatal communicative behaviors and data reproducibility. We found that there was a similar trajectory of USV changes between inbred and outbred mice, with both models displaying an increase in USV production after PD 4 that peaked on PD 12, followed by a significant reduction in USVs occurring on PD 16, with USVs ceasing on PD 20. At 4 of the 5 timepoints, the groups were statistically indistinguishable from one another, providing compelling evidence that results obtained in inbred mice are largely reproducible in outbred animals when the animals are of a similar background strain. However, on PD 12, the quantity of vocalizations emitted was significantly different between groups, with CD-1 mice producing significantly more USVs than FVB mice. This difference in USV production is best explained by the increased genetic complexity of outbred mice relative to inbred mice. Indeed, although CD-1 and FVB mice come from the same background strain, there is still a large degree of genetic variability between them, resulting in a congruent but not identical phenotype between models, accounting for the observed discrepancy. Overall, we found that there are minimal differences between inbred and outbred mice for the quantity of vocalizations produced, indicating that results should be reproducible between models.

When we compared the spectral and temporal characteristics of calls, we found numerous differences, with FVB mice vocalizing for a significantly longer duration on PD 4 and 8 and emitting calls of an overall higher pitch (minimum and maximum frequencies) than CD-1 mice, with no differences present between models for the loudness (mean amplitude) of the USVs. The differences in pitch and duration between models is notable, as alterations in both of these parameters are important constituents of numerous neurodevelopmental disease states. Specifically, murine models of conditions such as autism, epilepsy, or Cowden syndrome have reported fluctuations in call duration and pitch (3, 18, 38). Furthermore, when neonatal cries have been assessed in autistic infants, changes in duration and pitch have also been observed, indicating that these parameters are not only conserved across species, but may be significant indicators of neurodevelopment (13, 37). Additionally, when assessing the call type composition of USVs emitted from CD-1 or FVB mice, we similarly observed numerous differences between groups. Therefore, our data suggests that the spectral, temporal, and qualitative characteristics of early life vocalizations are more susceptible to the inherent differences in outbred and inbred mice than the production of USVs are.

We next assessed the inherent behavioral variability of both models, per day and overall. We found that CD-1 mice were less variable than FVB mice in the quantity of USVs emitted at 3 of the 4 timepoints. Moreover, when we assessed the total variability of each model, we found that CD-1 mice were more than 10% less variable than FVB mice. With respect to the spectral and temporal aspects of the vocalizations, we found that inbred and outbred mice were similarly variable, with there being no more than a 2% difference between groups for the total variability of the duration, maximum frequency, and minimum frequency of the calls, and only a 6% difference for the amplitude. Altogether, this indicates that outbred mice are as, if not less, variable than inbred mice across numerous measures. Importantly, our data also indicates that in measures that have more inherent variability (USV production), outbred mice are significantly less variable than their inbred counterparts, whereas in measures that are inherently less variable (call characteristics), inbred mice are only slightly less variable than outbred mice. Thus, CD-1 mice display an overall more favorable variability index than FVB mice, directly counteracting the common perception that outbred mice are more variable than inbred mice.

Interestingly, similar behavioral variability in outbred mice relative to inbred mice has been previously reported (27). Tuttle et al. (27) assessed adult mice and found that in 20 of 26 behavioral measurements outbred mice were as variable as their inbred counterparts, and perhaps even less variable. Therefore, in both neonates (our study) and adults (27), outbred animals have not been found to be any more variable than inbred animals. This is significant, since outbred mice better resemble the complexity of the human genome and thus display increased external validity relative to inbred mice. Additionally, outbred animals may also be more resistant to minute changes in experimental and environmental conditions than their inbred counterparts (27). Therefore, outbred mice have select advantages over inbred mice and, as our study suggests, fewer disadvantages than previously thought. Collectively, our study supports the growing body of research challenging the perception that outbred mice are significantly more variable than inbred mice (27, 41).

Future studies could expand upon the present work by assessing the variability of other outbred mouse strains relative to inbred strains. This would help to determine if all outbred animals are less variable and therefore more optimal for USV assessment than inbred mice or if CD-1 mice are particularly well-suited to assess communicative behaviors. Additionally, while our study assessed 5 timepoints at 4-day intervals, other studies could assess different timepoints (such as PD 2, 6, 10, 14) to garner a more comprehensive understanding of the subtle nuances between outbred and inbred mouse communicative behaviors. Studies could also assess and compare USV production in adult inbred and outbred mice to determine if increased genetic complexity has a more pronounced effect in mature animals. Altogether, numerous studies have assessed vocalizations and USV development in mice, however, more work needs to be performed if the potential of vocalizations is to be maximized (3, 21, 35, 42).

While the generation of USVs is a vital behavior in mice, there is also compelling evidence indicating that neonatal communicative behaviors are equally important in clinical populations. Specifically, Esposito et al. (43) assessed the crying behaviors of infants and observed that infants with autism are less likely to cry when the parent leaves the immediate environment, and will cry without a known cause. Infants with autism have also been reported to cry at a higher pitch, display shorter crying bouts, and to have an irregular loudness of their cries relative to neurotypical infants (11, 37, 44). While vocalizing behaviors have been mostly studied in clinical ASD populations, altered vocalizations have also been observed in other neurodevelopmental conditions such as tuberous sclerosis complex, epilepsy, and Tourette's syndrome, as well as in neurodegenerative conditions such as Alzheimer's disease and frontotemporal dementia (10–15). Therefore, compelling research is emerging which indicates that vocalizing behaviors, particularly during the neonatal period, have significant ramifications for human health and may constitute an early life behavioral biomarker for various disorders. Thus, the identification of optimal murine models that present with minimal behavioral variability will help to further elucidate vocalizing behaviors, constituting a necessary endeavor with clear applications to human health.

## Conclusion

Overall, our study assessed the consistency of USVs between inbred and outbred animals and was the first to assess the developmental trajectory of outbred mouse USVs relative to inbred mice, establishing a baseline of comparison for future studies. We found that both inbred and outbred animals from a Swiss background had the same USV developmental pattern and emitted approximately the same quantity of USVs on PD 4, 8, 16, and 20, indicating that USV results obtained in inbred mice should be similar to those obtained in outbred mice. However, there were numerous differences between outbred and inbred mice for the duration, minimum frequency, and maximum frequency of the USVs, as well as for each group's call type composition, suggesting that while USV production may be consistent between groups, the same is not necessarily true for other USV parameters. Importantly, we found that overall CD-1 mice displayed a more favorable variability index than FVB mice. Therefore, our study indicates that although inbred mouse models are valuable, their preferential use may not always be warranted nor necessarily ideal in all cases. Our study also indicates that additional studies need to be conducted that comprehensively examine the relative strengths and weaknesses of both inbred and outbred mice, as we found that common perceptions of models are not always accurate.

## Data Availability Statement

The original contributions presented in the study are included in the article/Supplementary Material, further inquiries can be directed to the corresponding author/s.

## Ethics Statement

The animal study was reviewed and approved by Yale University's Institutional Animal Care and Use Committee.

## Author Contributions

AB: research design and writing. HS: data analysis. MB: research design, experiment, data analysis, and writing. All authors contributed to the article and approved the submitted version.

## Conflict of Interest

The authors declare that the research was conducted in the absence of any commercial or financial relationships that could be construed as a potential conflict of interest.

## Publisher's Note

All claims expressed in this article are solely those of the authors and do not necessarily represent those of their affiliated organizations, or those of the publisher, the editors and the reviewers. Any product that may be evaluated in this article, or claim that may be made by its manufacturer, is not guaranteed or endorsed by the publisher.
