# Supplementary material for: CD-1 Outbred Mice Produce Less Variable Ultrasonic Vocalizations Than FVB Inbred Mice, While Displaying a Similar Developmental Trajectory
Source: Front Psychiatry. 2021 Aug 13;12:687060. doi: 10.3389/fpsyt.2021.687060 (PMC8407076; doi:10.3389/fpsyt.2021.687060)
Supplement: Supplementary file 1 [file Table_1.DOCX]

| **Table S1.** ANOVA *F* and *p* values | | | |  |
| --- | --- | --- | --- | --- |
| ANOVA analysis/  Parameters | Sex effect | Group by sex interaction | Day by sex  interaction | Day by sex by group interaction |
| USV production | *F*_1,56_ = .16  *p* = .69 | *F*_1,56_ = 1.43  *p* = .24 | *F*_4,117_ = .36  *p*= .84 | *F*_4, 224_ = 1.29  *p* = .28 |
| USV duration | *F*_1,39_ = .65  *p* = .42 | (*F*_1,39_ = .89  *p* = .35) | *F*_3,117_ = 1.55  *p* = .20 | *F*_3,117_ = .10  *p* = .96 |
| Minimum frequency | *F*_1,39_ = 1.40  *p* = .24 | *F*_1,39_ = .65  *p* = .43 | *F*_3, 117_ = .85  *p* = .47 | *F*_3, 117_ = .19  *p* =.90 |
| Maximum frequency | *F*_1,39_ = 1.14  *p* = .29 | *F*_1,39_ = .63  *p* = .43 | *F*_3,117_ = .47  *p* = .70 | *F*_3,117_ = .67  *p* =.57 |
| Mean amplitude | *F*_1,39_ = .34  *p* =.56 | *F*_1,39_ = 2.97  *p* =.09 | *F*_3,117_ = 1.19  *p* = .32 | *F*_3,117_ = .29  *p* = .83 |
| Weight | *F*_1,56_ = .11  *p* = .74 | *F*_1,56_ = 1.87  *p* = .18 | *F*_4,224_ = 1.84  *p* = .14 | *F*_4,224_ = 1.76  *p* = .19 |
